# Whole human genome proteogenomic mapping for ENCODE cell line data: identifying protein-coding regions

**DOI:** 10.1186/1471-2164-14-141

**Published:** 2013-02-28

**Authors:** Jainab Khatun, Yanbao Yu, John A Wrobel, Brian A Risk, Harsha P Gunawardena, Ashley Secrest, Wendy J Spitzer, Ling Xie, Li Wang, Xian Chen, Morgan C Giddings

**Affiliations:** 1 College of Arts and Sciences, Boise State University, Boise, ID, USA; 2 Department of Biochemistry & Biophysics, UNC School of Medicine, Chapel Hill, NC, USA; 3 Program in Molecular Biology & Biotechnology, UNC School of Medicine, Chapel Hill, NC, USA

**Keywords:** Proteogenomic mapping, MS/MS spectra, Genome annotation, Proteomics, Genomics

## Abstract

**Background:**

Proteogenomic mapping is an approach that uses mass spectrometry data from proteins to directly map protein-coding genes and could aid in locating translational regions in the human genome. In concert with the ENcyclopedia of DNA Elements (ENCODE) project, we applied proteogenomic mapping to produce proteogenomic tracks for the UCSC Genome Browser, to explore which putative translational regions may be missing from the human genome.

**Results:**

We generated ~1 million high-resolution tandem mass (MS/MS) spectra for Tier 1 ENCODE cell lines K562 and GM12878 and mapped them against the UCSC hg19 human genome, and the GENCODE V7 annotated protein and transcript sets. We then compared the results from the three searches to identify the best-matching peptide for each MS/MS spectrum, thereby increasing the confidence of the putative new protein-coding regions found via the whole genome search. At a 1% false discovery rate, we identified 26,472, 24,406, and 13,128 peptides from the protein, transcript, and whole genome searches, respectively; of these, 481 were found solely via the whole genome search. The proteogenomic mapping data are available on the UCSC Genome Browser at http://genome.ucsc.edu/cgi-bin/hgTrackUi?db=hg19&g=wgEncodeUncBsuProt.

**Conclusions:**

The whole genome search revealed that ~4% of the *uniquely* mapping identified peptides were located outside GENCODE V7 annotated exons. The comparison of the results from the disparate searches also identified 15% more spectra than would have been found solely from a protein database search. Therefore, whole genome proteogenomic mapping is a complementary method for genome annotation when performed in conjunction with other searches.

## Background

The human genome holds many secrets – the deeper we peer, the more we uncover. In 2003, the National Human Genome Research Institute (NHGRI) launched a pilot project called the ENCyclopedia Of DNA Elements (ENCODE) to analyze 44 euchromatic regions of the human genome. The pilot project revealed surprising results, such as pervasive intragenic and intergenic transcription, new intronic and intergenic exons, overlapping transcripts, and distant transcriptional start sites, challenging the conventional model of genes and their transcription [1].

Following these successes, the NHGRI expanded ENCODE to study the entire human genome, to provide the scientific community with a comprehensive list of functional elements including protein-coding and non-coding transcripts, transcriptional regulatory regions, histone marks, and more. In the production phase, the ENCODE Consortium produced deep data via extensive high-throughput experiments in combination with both novel and existing computational techniques [2,3].

Despite these efforts, the transcribed regions of the genome that are translated into proteins, versus those that serve some other role, remain elusive. GENCODE, a sub-project of ENCODE, has performed an exhaustive manual annotation of the human genome to identify protein-coding transcripts, and though this is likely the most comprehensive human genome annotation to date, the evidence for protein-coding capacity has come mostly from indirect sources, not from the measurement of proteins themselves. About 50% of human transcripts are classified as non-protein-coding [2]. While many do not resemble known protein-coding transcripts, some do not appear to be non-coding functional RNAs either, hence their roles remain unclear.

Proteogenomic mapping is a process that has been used for various organisms to help identify protein-coding regions and transcripts, by mapping mass spectrometry (MS) data from biologically-derived proteins directly to genomic and/or transcript sequences [4-8]. This approach has been used to identify new genes, new alternative splice variants, new translational start sites, new upstream open reading frames (ORFs), and has also been used to classify pseudogenes as protein-coding [4-6,8-17]. For instance, Menon *et al.* conducted a large-scale analysis of MS data from the plasma proteome of a mouse model of human pancreatic cancer. The study employed a non-redundant database containing a 3-frame translation of Ensembl transcripts and gene models from the ECgene database, which identified 92 novel protein variants [14]. Recently, Brosch *et al.* performed proteogenomic mapping using Augustus-predicted transcripts from the mouse genome. They discovered 10 novel protein-coding genes, novel alternative splice forms for 53 genes, and classified 9 pseudogenes as protein-coding [9].

**Figure 1:**
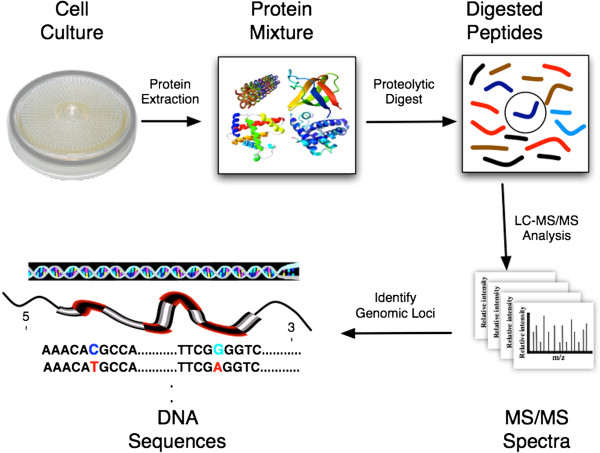
**Overview of bottom-up proteomics and proteogenomic mapping.** After cell lysis, proteins are extracted from a biological sample and are proteolytically digested into peptides. The peptide mixture is commonly separated by liquid chromatography and introduced into a tandem mass spectrometer, which produces MS/MS spectra. The resulting spectra are matched against an *in silico* translation and proteolytic digestion of genomic DNA sequences in all six reading frames to identify peptides. The matched peptides are then mapped back to the DNA sequences to identify the genomic loci for the analyzed proteins.

Bottom-up proteomics is the most widespread means of proteogenomic mapping. Briefly, cells are collected or cultured then lysed, often followed by subcellular fractionation. Proteins are extracted then cleaved proteolytically into peptides, either by direct in-solution digestion, or after gel-based separation followed by in-gel digestion. The proteolytic peptides are separated to reduce sample complexity before introduction into the mass spectrometer [18]. In tandem MS (MS/MS), the mass spectrometer measures the mass-over-charge (m/z) of each peptide ion, sequentially breaks it along the peptide backbone, then measures the m/z of the resulting pieces. The series of fragment masses provides a signature that can then be used to identify the peptide from a database search [19-25] or by *de novo* sequencing [26,27]. When the reference database used to identify peptides (and therefore proteins) contains DNA sequences (genome, transcripts, predicted transcripts, etc.), the process is termed *proteogenomic mapping*[8] (Figure 1)*.*

Prior proteogenomic mapping efforts of the human genome relied primarily on databases of putative ORFs, full-length cDNAs, or a combination of various predicted transcripts [11,16,17,28]. However, our ability to correctly predict protein-coding transcripts is limited, and hence the approaches that rely on predictions may also be limited. To our knowledge, the most recent human genome proteogenomic work was done by Bitton *et al.,* which identified 346 novel peptides at a 5% FDR [29]. However, they first performed a series of pre-screening searches which dramatically reduced the database size before beginning the human genome proteogenomic mapping process.

Whole genome proteogenomic mapping is an alternative approach that does not rely on transcript or gene prediction. It has the drawback that the larger genome database reduces sensitivity, yet it has one significant strength: its ability to find putative protein-coding exons outside of known or predicted genic regions. As such, it can be seen as a complementary method to protein or transcript database searches: the methods performed in conjunction with one other will likely yield maximal coverage of the proteo-genome. The applications and challenges of proteogenomic mapping have been reviewed in a recent publication [30].

In this manuscript, we describe an effort to perform proteogenomic mapping of the human genome as part of the ENCODE project. We produced proteomic data using the ENCODE Tier 1 cell lines K562 and GM12878. Proteins from each cell line were derived via front-end sample preparation protocols including subcellular fractionation, GELFREE fractionation [31], filter-aided sample preparation (FASP) [32], and microwave-assisted tryptic digestion [33]. Peptides were analyzed on an LTQ Orbitrap Velos mass spectrometer (Thermo Scientific) to produce ~1 million high-resolution MS/MS spectra. We mapped these spectra against the UCSC hg19 whole human genome, and against GENCODE V7 protein and transcript databases, and then compared the results from all three searches to identify the best-matching peptide for each spectrum. This comparison increased the confidence of the identification of the putative new protein-coding exons found from the whole genome search and also augmented the total number of spectral identifications.

## Results

We performed *shotgun* proteomic analyses for two ENCODE Tier 1 cell lines and mapped the resulting 998,570 MS/MS spectra against the GENCODE V7 protein and transcript databases, as well as the whole human genomic sequence (UCSC hg19). We then compared the results from all three searches to identify the best-matching peptide for each spectrum. The complementary nature of this comparative analysis provided confidence for the identification of non-exonic peptides located outside the GENCODE V7 annotation, in addition to identifying 15% more spectra than would have been identified solely from a protein database search.

### GENCODE V7 protein and transcript search results

**Figure 2:**
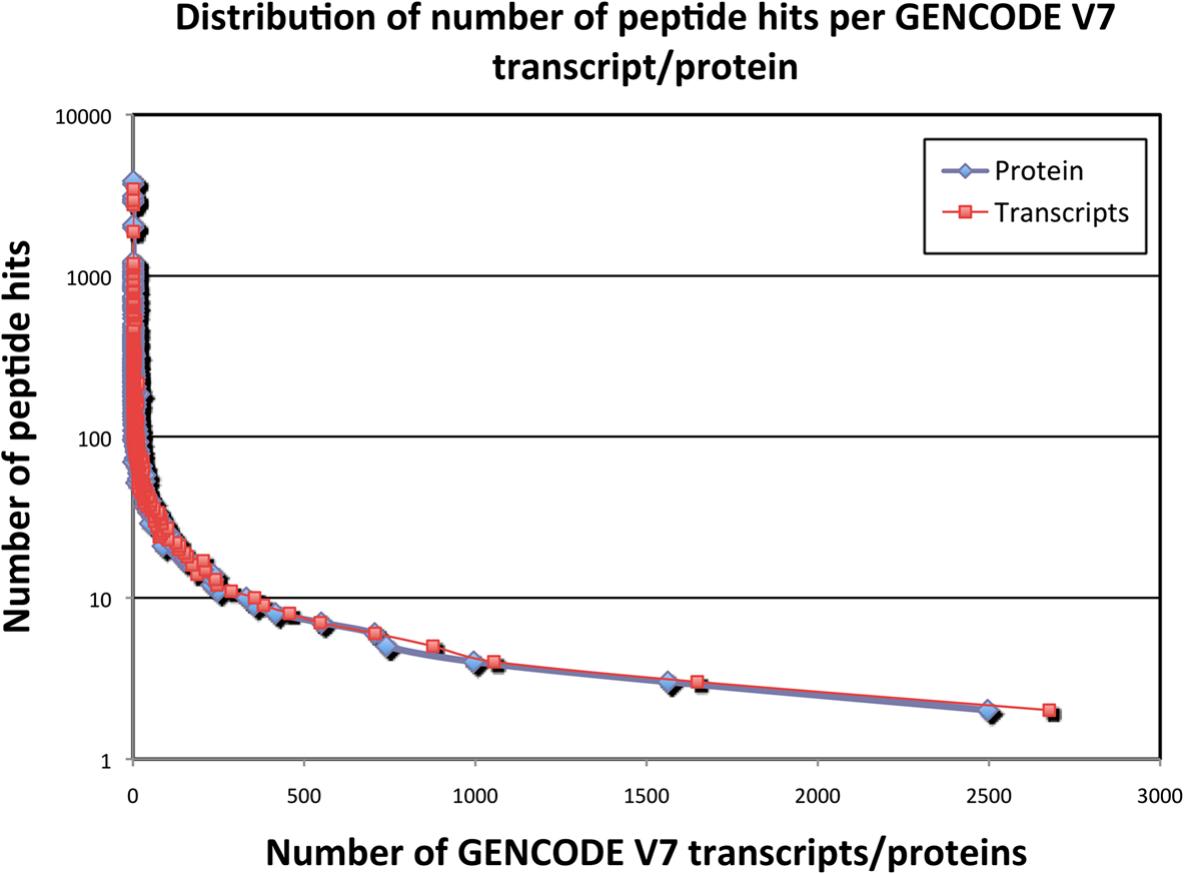
**The distribution of the number of peptide hits per protein/transcript.** The x-axis represents the number of protein/transcripts and the y-axis represents the number of peptides that matched to that number of protein/transcripts. Only proteins/transcripts matched to 2 or more peptides are considered in the distribution. The points in blue represent the peptide hits from the GENCODE V7 annotated proteins, while the red points represent those from the GENCODE V7 annotated transcripts.

We searched the 998,570 MS/MS spectra against the GENCODE V7 annotated protein set. We enzymatically digested each of these proteins *in silico* and scored the resulting peptides against each MS/MS spectrum using the HMM_Score algorithm [22]. The search resulted in identifying 20,051 proteins from 26,591 distinct peptides matching to 115,164 MS/MS spectra, at a 1% false discovery rate (FDR) (Table 1). The distribution of peptide identifications for these proteins is shown in Figure 2.

**Table 1:** Total spectra searched and identified from whole genome, GENCODE V7 transcript and protein searches

**Total spectra searched**	**Databases (size)**	**# of distinct spectra mapped (# of distinct spectra mapped when only best match considered)**	**# of distinct peptides identified (# of distinct peptides when only best match considered)**	**# of distinct genomic loci/proteins/transcripts identified (# of distinct loci/transcripts/proteins when only best match considered)**
998570	whole genome (~3.2 GB)	62308 (62218)	13143 (13128)	16832 (16808)
998570	GENCODE V7 transcript (~200 MB)	111138 (110738)	24503 (24406)	21032 (20985)
998570	GENCODE V7 protein (~44 MB)	115164 (114618)	26591 (26472)	20051(20013)

Results presented are at a 1% FDR. The bracketed numbers represent the number of identifications after comparing the results from the three searches and keeping only the best match.

We also performed proteogenomic mapping against GENCODE V7 annotated transcripts using the same set of spectra. We performed a 3-frame translation of 84,408 GENCODE V7 transcripts (which only included mRNAs) and constructed a protein database where each stop codon represented the end of one protein and the beginning of another. We then enzymatically digested those proteins *in silico* and scored the resulting peptides against each MS/MS spectrum. The search resulted in finding translational evidence for 21,032 transcripts, by identifying 24,503 distinct peptides from 111,138 MS/MS spectra, at a 1% FDR (Table 1 and Figure 2).

### Whole human genome search results

In whole genome proteogenomic mapping, spectra are matched to peptides produced from an *in silico* translation and proteolytic digestion of genomic sequences in all six reading frames [7]. The matched peptides are then mapped back to the DNA sequences to identify the genomic loci for the analyzed proteins. The whole genome search identified 13,143 distinct peptide sequences, matching to 62,308 MS/MS spectra, at an estimated 1% FDR. These peptides corresponded to 16,832 distinct genomic loci. Because many different spectra can match to a single peptide, and because a given peptide sequence can reside in different places in the genome, the number of peptides and the number of genomic loci differ from the number of spectra.

### Comparison of GENCODE V7 protein, transcript, and hg19 whole genome search results

One of the goals of this study was to explore what percentage of proteins may be missing from the current protein database annotation, and therefore how many additional MS/MS spectra could be identified from an unbiased, whole human genome proteogenomic mapping effort. To increase the confidence of all identifications, we compared the results from the three different searches and identified the best-matching peptide for each MS/MS spectrum, regardless of which search yielded that best peptide-spectrum match (PSM). For a given spectrum, if two different best-ranking peptides from two different databases were identified, then the PSM with the highest HMM_Score was taken as the ‘correct’ identification, and the others were removed from the results.

This comparison resulted in finding 1,036 spectra that matched to different top-ranking peptides in the different databases. Scrutiny of these results revealed that 546 spectra identified from the protein database search had better matches from either the genome or the transcript searches, and 400 spectra from the transcript search had better matches from either the genome or the protein database searches. Similarly, 90 spectra identified from the genome search had better matches in the protein or transcript database searches.

We considered only the best-matching peptides from all three searches, i.e., those retained after removing the 546, the 400 and the 90 spectral hits from the protein, transcript and genome searches, respectively. After removal, the GENCODE V7 protein search identified 26,472 distinct peptides belonging to 20,013 proteins from 114,618 MS/MS spectra; the transcript search identified 24,406 distinct peptides belonging to 20,985 transcripts from 110,738 MS/MS spectra; and the whole genome proteogenomic search identified 13,128 peptides from 62,218 MS/MS spectra corresponding to 16,808 distinct loci (Table 1). The combination of the results of all three searches identified 28,530 peptides from a total of 131,586 MS/MS spectra, at a 1% FDR. The combination and comparison of results identified 16,968 additional MS/MS spectra and 2,058 additional peptides which would not have been found from a protein database search alone.

**Figure 3:**
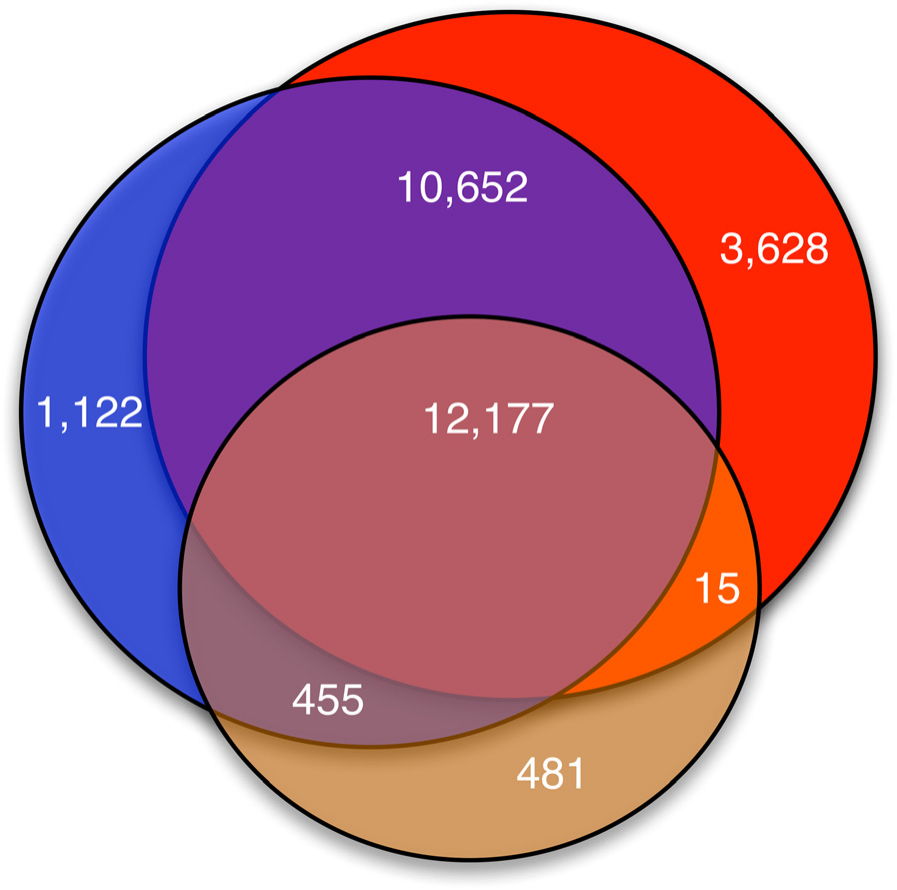
**Venn diagram of distinct peptide identifications from the protein, transcript, and whole genome searches.** The deep red segment in the center represents the 12,177 peptides identified from all three searches. The segment in red represents the 3,628 peptides identified solely from the GENCODE V7 protein search; the blue segment represents the 1,122 peptides identified solely from the GENCODE V7 transcript search; and the brown segment represents the 481 peptides identified solely from the whole genome search.

When we performed a cross comparison, the same 12,177 unique peptides were identified from all three searches. There were 3,628 best-matching peptides identified solely from the protein database search, 1,122 identified solely from the transcript search, and 481 identified solely from the whole genome search. A Venn diagram of these peptide identifications is shown in Figure 3, which shows that 1,577 peptides were identified from the transcript search but were not identified from protein database search. We closely examined these 1,577 identified peptides to ascertain whether they were due to frame shift or non-coding transcript translation. We found that 77 of 1,577 (~5%) identified peptides were products of frame shifting, while 313 (~20%) were due to the translation of non-coding transcripts. The remaining 1,187 (~75%) peptides belong to novel alternative spliced forms of known protein-coding transcripts, and were located in untranslated regions (UTRs) or in UTR-exon boundary regions.

For the purpose of this investigation, we believed the best-matching peptide should be taking as the ‘correct’ identification, though within the 1% FDR, occasionally the peptide identified by the algorithm as the second- or third-best match may actually represent the ‘correct’ match. To mitigate this possibility, we kept the FDR appropriately conservative (1%); however, we acknowledge the fact that the peptide ranked highest by the algorithm may not always represent the ‘correct’ identification.

### Analysis of unique proteogenomic matches against wgEncodeGencodeCompV7

To provide a more precise picture of what can be gained from a proteogenomic search, we focused on our *unique matches* – matches for which the identified peptide appears at only one site in the genome. The subset of unique matches was composed of 48,012 distinct MS/MS spectra, which matched to 11,540 unique peptide sequences, hence 11,540 genomic loci. Several spectra matching to the same peptide lends extra support for the validity of the match, and can be used as an approximate relative quantitative measure of protein abundance [34]. We uploaded our uniquely mapping proteogenomic results from the whole genome search as a custom track to the UCSC Table Browser to compare them against GENCODE V7 annotated genes.

When these 11,540 unique peptides were compared against the GENCODE V7 annotation, 11,120 were found to be exonic and the remaining 420 were non-exonic. In this paper, we take the terms ‘exonic’ and ‘non-exonic’ to mean exonic/non-exonic according to the GENCODE V7 annotation. We performed an analysis and found that of those 420 non-exonic matches, 72 corresponded to intronic regions and 348 corresponded to intergenic regions.

**Figure 4:**
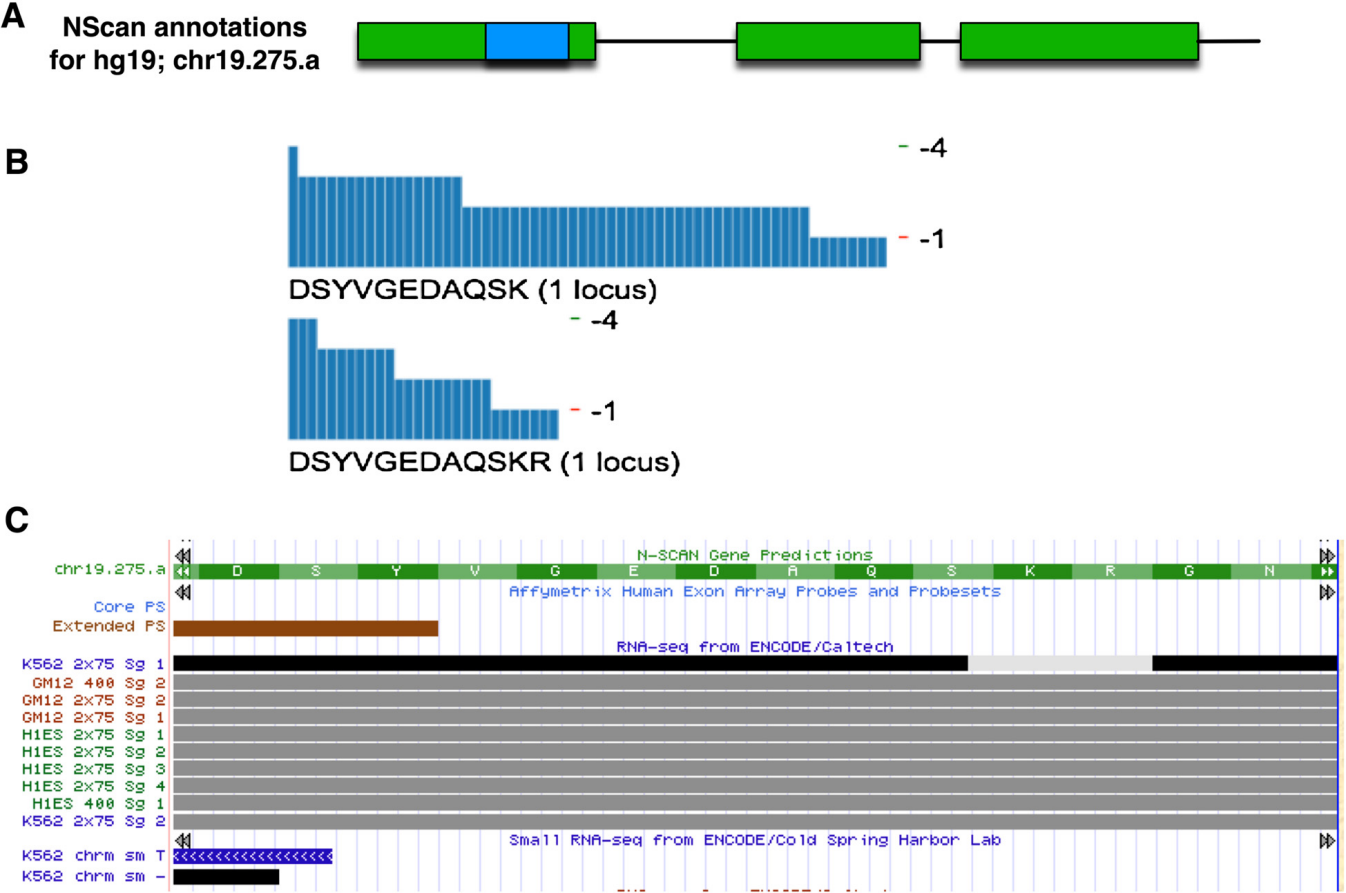
**An example of unique GENCODE V7 intergenic proteogenomic matches.** Panel **A** shows that these unique proteogenomic matches overlap with a protein-coding exon predicted by NScan. Blue boxes represent proteogenomic matches, green boxes represent predicted protein-coding exons, and black lines represent introns. Panel **B** summarizes the total MS/MS spectral support for each of the two matches in this region, where each vertical dark blue bar represents a distinct spectral match for the same peptide, with the height of the bar showing the E-value for the identification (E-values ranging from 1.0×10^-1^ to 1.0×10^-4^). More and/or taller bars indicate stronger support. Panel **C** shows ENCODE/Caltech RNA-Seq evidence and other transcriptional data for the same region. Both matches are identified from multiple spectra, indicating relatively strong support.

Figure 4 shows unique proteogenomic mapping matches outside the GENCODE V7 annotation. The location was identified from multiple MS/MS spectra from two distinct precursor m/z sets. The same location has RNA-Seq evidence from ENCODE/Caltech.

### Other evidence related to unique, non-exonic matches

The 420 unique, non-exonic matches could represent new genic regions, new isoforms of known genes, or false discoveries that fall within the 1% FDR. We attempted to determine whether there was other supporting evidence for these matches by searching for expression data and predicted exons.

Using the UCSC Table Browser, we examined all unique, non-exonic matches for evidence of transcriptional activity and/or predicted exons, using: HAIB RNA-Seq data (wgEncodeHaibRnaSeqA549Dex100nm RawRep1); GENSCAN gene predictions (genscan); Human ESTs (all_est); Burge RNA-Seq data (burgeRnaSeq GemMapperAlignBT474); Ensembl exons (acembly); UW Affy Exon Array data (wgEncodeUwAffyExonArray Gm12878SimpleSignalRep1v2); and Duke Affy Exon Array data (wgEncodeDukeAffyExonGm12878SimpleSig nalRep1). Information about each of these datasets can be found with their individual tracks on the UCSC Genome Browser. Of the 420 unique, non-exonic matches, 268 overlapped with the HAIB RNA-Seq data; 215 overlapped with GENSCAN predicted exons; 175 overlapped with human ESTs; 120 overlapped with Burge RNA-Seq data; 281 overlapped with Ensembl exons; 196 overlapped with WU Affy Exon Array data; and 221 overlapped with the Duke Affy Exon array data (Table 2).

**Table 2:** Unique GENCODE V7 non-exonic peptides and their overlap with different expression data and predicted exons

**Total non-exonic peptides**	**Data types**	**Number of distinct peptides that overlap**
420	HAIB RNA-Seq	268
	GENSCAN gene predictions	215
	Human ESTs	175
	Burge RNA-Seq	120
	Ensembl exons	281
	UW Affy Exon Array	196
	Duke Affy Exon Array	221

Results presented are at a 1% FDR. Information about each dataset can be found with its individual track on the UCSC Genome Browser.

The union of intersection between our unique non-exonic matches and all seven datasets is 368, i.e., each of these 368 unique hits had at least one other piece of supportive evidence, either transcriptional evidence or predicted exons. Similarly, the central intersection from all seven datasets, i.e. the matches for which all seven datasets overlapped, was 14. The results indicate that ~88% (368/420) of our unique proteogenomic matches were supported by either predicted exons or by the presence of transcriptional activity. When we examined the remaining 52 matches for which there was no corroborating evidence, we found that 3 were intronic and 49 were intergenic.

### Proteogenomic tracks to UCSC genome browser

**Figure 5:**
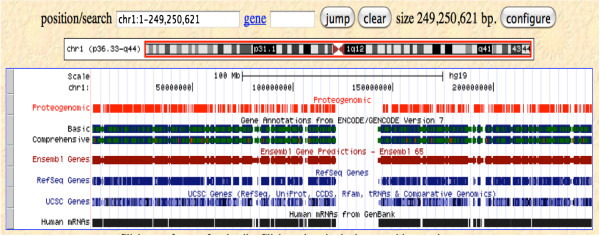
**A UCSC Genome Browser screenshot showing proteogenomic coverage across chromosome 1, with several annotation sets.** The red line at the top represents our proteogenomic matches. The annotation sets shown here include GENCODE V7, Ensembl, RefSeq, and the UCSC annotation. The black line at the bottom shows the human mRNAs from GenBank.

To facilitate the interpretation of proteogenomic data within a genomic context, we produced UCSC bed tracks. The ENCODE proteogenomic tracks were submitted to the ENCODE Data Coordination Center (DCC) at UCSC in accordance with ENCODE data standards. Though only the best-matching peptides at a 1% FDR are presented in this manuscript, the uploaded results include first-, second- and third-ranked peptides at a 5% FDR, to conform to ENCODE standards. The tracks can be browsed to see where and how proteogenomic data line up with other types of evidence, such as human ESTs, RNA-Seq, etc. (Figures 4 and 5), and can be accessed at http://genome.ucsc.edu/cgi-bin/hgTrackUi?db=hg19&g=wgEncodeUncBsuProt. Figure 5 shows a UCSC Genome Browser screen shot illustrating the alignment between our proteogenomic mapping loci and several other annotation sets for chromosome 1.

## Discussion

We produced MS/MS spectra from two ENCODE Tier 1 cell lines and searched them against GENCODE V7 annotated protein and transcript sets, as well as against the standard human genome sequence (UCSC hg19). To achieve as complete proteomic coverage as possible, we used spectra from two cell lines, rather than from a single line: this approach provided us with ~1 million high-quality spectra to facilitate large-scale proteogenomic analysis. We also employed a combination of strategies to increase the coverage of the analyzed proteins, such as filter-aided sample preparation, microwave-assisted in-filter digestion, and subcellular fractionation. We also used a state-of-the-art Eksigent Ultra-LTQ Orbitrap mass spectrometer which improved the accuracy of mass measurements and provided a more complete fragmentation pattern.

In addition, we compared the results from the three different database searches to identify which PSM from which search was scored highest by the HMM_Score algorithm. We found that though whole genome mapping is a less sensitive method, it identified 481 putative novel peptides because they do not belong in annotated exons. These peptides could come from protein isoforms whose corresponding mRNAs have either not yet been captured in an expression database, or whose mRNAs are not currently annotated as protein-coding. These results indicate that a search using a set of annotated transcripts or a standard protein database may miss crucial supporting evidence for new alternative splices and possibly for unannotated genes. Performing proteogenomic mapping using both transcript and whole genome sequences identified ~15% more MS/MS spectra than would have been found solely by the protein database search. These searches are complementary: when performed in conjunction with one another, they improve the total coverage of proteomic identifications.

We uploaded browsable bed files to the UCSC Genome Browser, which offers a unique opportunity to inspect proteomic data within the context of other genomic data. From the alignment between our proteogenomic mapping results and different annotation sets, researchers can now identify which annotated protein-coding transcripts have confirmatory protein evidence, or if any sequences annotated as introns act as protein-coding exons in some disease states. These new proteogenomic mapping tracks could help researchers answer many other questions that could not otherwise be addressed without direct protein evidence.

### Future applications of human genome proteogenomic mapping work

Proteogenomic mapping has been used previously to aid in human genome annotation [11,16,17,28]. Whole genome mapping could also be used to further explore many of the unexpected results that have been found using large transcriptional databases. For example, there is evidence that a large number of human cDNAs have an upstream start codon (ATG) preceding the start codon of the longest known ORF [35-37]. A serial analysis of gene expression tags revealed that antisense transcripts are far more widespread than previously known [38]. In addition, the ENCODE Consortium found many intergenic, antisense, and chimeric transcripts [1,3].

These novel transcripts require further study to determine whether they encode proteins. Performing an unbiased whole genome proteogenomic mapping could provide support for the translation of small ORFs, antisense transcripts, non-coding RNAs, or sites annotated as introns [39]. Whole genome proteogenomic mapping could also aid in biomarker discovery as aberrant splice isoforms and amplicons are known to be associated with many cancers [40-42].

### Limitations of whole genome proteogenomic mapping

Whole genome proteogenomic mapping can offer new insights about the translational regions of the human genome; however, the method has some limitations. First, a whole human genome search reduces sensitivity and specificity due to the increased size of the database. Furthermore, incorporating every splice site in an unbiased manner (i.e. between every GT-AG) and considering post-translationally modified peptides would create an unmanageably large database, ultimately increasing false positive assignments.

Moreover, protein expression depends on different cellular and developmental conditions, as well as different cell types [43]. We used only one standard genomic sequence (UCSC hg19) and two different cell cultures not directly related to that genome. Single nucleotide polymorphisms, copy number variants, and other genetic differences exist between individuals, which produce different proteomic profiles. Minor sequencing errors could produce different theoretical proteomic profiles, affecting the correctness of the PSMs identified. Both Tier 1 cell lines are cancer-derived, which may present additional somatic mutations, further complicating protein expression. Therefore, additional proteomic analyses are needed which consider different cellular, developmental and genetic variations, as well as different cell types.

## Conclusions

In summary, we mapped proteomic data against three different databases (protein, transcript, and whole human genome) to confidently identify putative new translational regions of the human genome, and thereby increase the total proteomic coverage. We used a very tight precursor mass tolerance (0.02 Da) and an FDR of 1% to reduce the chance of false positive identifications. The comparison of search results found that ~4% of the peptides identified from the whole genome search were outside GENCODE annotated exons; the approach also identified ~15% more spectra than would have been identified solely from a protein database search.

At present, whole genome proteogenomic mapping offers the opportunity to identify peptides that would not be found solely from a protein database search. However, whole human genome proteogenomic mapping is still in its infancy and its current value is only in pinpointing new genomic areas of focus. As improvements are made in mass spectrometry and computer technologies, and once different cellular/developmental conditions and genetic variations are considered, we speculate that proteogenomic mapping, performed in conjunction with other database searches, could significantly increase knowledge about the translational regions of the human genome.

## Methods

### Mass spectrometry data generation

#### Cell culture, subcellular fractionation, and sample preparation

Human ENCODE cell lines K562 and GM12878 were cultured in Roswell Park Memorial Institute Medium 1640, supplemented with 10% fetal bovine serum, penicillin (100 units/ml), and streptomycin (100 mg/ml). Cells were maintained in a humidified incubator with 5% carbon dioxide at 37°C.

Subcellular fractionation was performed on both cell lines following a common protocol, producing nuclear, mitochondrial, cytosolic, and membrane fractions [44]. For SDS-PAGE separation and in-gel digestion, a standard procedure was followed [45]. For GELFrEE separation, a GELFREE 8100 Fractionation System (Protein Discovery, Knoxville, TN) was used according to the manufacturer’s protocol. The collected protein fractions were further processed using filter-aided sample preparation (FASP) [32] or the GOFAST method [33].

### RPLC-MS/MS analysis

Reversed Phase Liquid Chromatography (RPLC) MS/MS analysis was performed on a nanoLC-Ultra system (Eksigent, Dublin, CA) coupled with an LTQ Orbitrap Velos mass spectrometer (Thermo Scientific, San Jose, CA). ProteoPep™ II C18 column (75 μm × 15 cm, 300 Å, 5 μm, New Objective, MA) and linear gradient was run from 100% buffer A (0.1% formic acid in H_2_O) to 40% buffer B (0.1% formic acid in ACN) in 150 minutes, and then to 80% buffer B for another 30 minutes. Eluted peptides were ionized and analyzed in a data-dependent manner using XCalibur software (version 2.1, Thermo Scientific). The top five most abundant precursor ions were selected for further MS/MS analysis. Collision-induced dissociation (CID) was used to fragment peptides and then each fragment’s m/z was measured.

### Data sets produced

We produced MS/MS spectra for four fractions (nuclear, mitochondrial, cytosolic, and membrane) of both cell lines K562 and GM12878 using SDS-PAGE and GELFrEE. The spectra from the GM12878 cytosolic fraction did not meet quality control standards, so we omitted that fraction from our searches. MS/MS spectra from a duplicate membrane fraction of cell line K562 was also generated using the GOFAST method. Therefore, we obtained eight different sets of data totaling 998,570 MS/MS spectra. All MS/MS spectra in dta format and the proteogenomic mapping results were uploaded to Proteome Commons, accessible via http://giddingslab.org/data/encode/proteome-commons.

### Proteogenomic mapping

#### Databases used

We performed proteomic searches against the GENCODE V7 translated protein set, consisting of 84,408 annotated protein sequences . We also used a 3-frame translation and proteolytic digestion of the GENCODE V7 annotated transcripts (mRNA of 84,408 annotated protein sequences). Both of these sequences were annotated by the Wellcome Trust Sanger Institute and are available at http://www.gencodegenes.org/releases/7.html. The database sizes for the protein and transcript databases were ~44 MB and ~200 MB, respectively.

We also used a 6-frame translation and proteolytic digestion of the whole human genome for our proteogenomic mapping (UCSC hg19, 2009, available at http://hgdownload.cse.ucsc.edu/goldenPath/hg19/chromosomes/), resulting in a database size of ~3.2 GB.

### Mapping procedure

We used the newly developed Peppy to perform all searches. Peppy is an integrated software capable of processing the whole human genomic sequence in a single run, as well as protein and transcript databases [Risk B and Giddings MC: Peppy: an all-in-one tool for proteogneomic searching of MS/MS spectra. Manuscript in preparation]. For the whole genome search, Peppy performed an *in silico* 6-frame translation and proteolytic digestion of DNA sequences to create a database ‘on the fly’. For all searches, we used the HMM_Score algorithm to match and score peptides to spectra [22]. A common proteomic search engine such as Mascot or Sequest could have been used to match and score peptides to spectra; however neither program was designed to easily handle a six-frame translation/digestion and search of a whole human genome.

For all searches, we used a precursor mass tolerance of 0.02 Da, a fragment mass tolerance of 0.5 Da, allowed one missed cleavage for tryptic digestion, chose mono-isotopic masses for amino acids, and did not consider modifications. The E-value was calculated for each PSM using the method described by Fenyö *et al.*[46]. The decoy databases were derived from the original databases (target databases) by reversing the target sequences for each of the three databases. The E-value threshold for each search was calculated for a 1% FDR using the decoy database search according to Kall *et al.*[47].

The thresholds for a specific FDR were calculated for each database individually; these separate calculations were necessary to create FDRs that were meaningful for the varying sizes of each database. For example, the database resulting from the 6-frame translation and digestion of the human genome was more than 1000 times as large as that of the protein database, therefore the FDR threshold for the human genome search was higher than that of the protein database. It is important to note that though the thresholds differed based on database size, the resulting FDR was 1% for all three searches.

## Abbreviations

PSM: Peptide-spectrum match; ORF: Open reading frame; UTR: Untranslated region; MS: Mass spectrometry; MS/MS: Tandem mass spectrometry; m/z: Mass-over-charge; FDR: False discovery rate; ENCODE: Encyclopedia of DNA elements; UCSC DCC: University of California Santa Cruz data coordination center; FASP: Filter-aided sample preparation; RPLC: Reversed-phase liquid chromatography; CID: Collision-induced dissociation.

## Competing interests

The authors declare that they have no competing interests.

## Authors’ contributions

JK conceived the project, conducted all analyses, and drafted the manuscript. YU, HPG, LW, and LX performed the MS/MS analysis and were advised by XC. JAW ran the MS/MS spectral data using Peppy, the software developed by BAR. WJS was the primary editor of the manuscript (with minor assistance by AS), and MCG directed the project. All authors read and approved the final manuscript.
